# Brain Health Services for the prevention of dementia: aligning pilot experiences for clinical deployment

**DOI:** 10.1002/alz.091339

**Published:** 2025-01-09

**Authors:** Federica Ribaldi

**Affiliations:** ^1^ Laboratory of Neuroimaging of Aging (LANVIE), University of Geneva, Geneva, Switzerland; Geneva Memory Center, Department of Rehabilitation and Geriatrics, Geneva University Hospitals, Geneva Switzerland

## Abstract

**Background:**

The European Task Force for Brain Health Services (BHS) has developed the concept for evidence‐based and ethical services targeting individuals with intact cognition and a high risk of dementia. BHS are complementary to current memory clinics and their missions consists of four pillars: risk assessment and profiling, risk communication, personalised risk reduction, and cognitive enhancement. Recommendations for the deployment of the four pillars have been published, and pilot BHS experiences are underway in European countries and the United States. The field is now ripe to draw lessons from these pilot experiences and move forward towards routine clinical deployment.

**Method:**

Experts specializing in the four pillars are collaborating to assess the opportunities and challenges associated with implementing BHS in clinical practice, drawing on lessons from ongoing pilot programs. Additionally, experts in health economics are developing a business plan for the practical deployment of BHS in Geneva, along with a European business case. The BHS task force is actively formulating a comprehensive roadmap for clinical deployment, accounting for anticipated advancements over the next 3‐5‐10 years, including the incorporation of blood‐based biomarkers for risk profiling, personalized prevention and cognitive enhancement through pharmacological and non‐pharmacological interventions (e.g., non‐invasive brain stimulation).

**Result:**

Practical programs and protocols for implementing BHS in different environments and countries will be presented ad AAIC 2024. Furthermore, we will present the roadmap for the clinical deployment of each service, along with expected timelines and the business plan for the setup of BHS as hybrid or stand‐alone services, emphasizing the progress made in aligning pilot experiences and the potential impact on dementia prevention.

**Conclusion:**

The development and widespread diffusion of BHS in Europe and USA will contribute to fighting dementia and reduce its burden on patients, their families, and society.

